# The anti-shigellosis activity of the methanol extract of *Picralima nitida* on *Shigella dysenteriae* type I induced diarrhoea in rats

**DOI:** 10.1186/1472-6882-13-211

**Published:** 2013-08-17

**Authors:** Laure Brigitte Mabeku Kouitcheu, Joseph Lebel Tamesse, Jacques Kouam

**Affiliations:** 1 Microbiology and Pharmacology Laboratory, Department of Biochemistry, Faculty of Science, University of Dschang-Cameroon, P. O. Box 67, Dschang, Cameroon; 2 Department of Biological Sciences Higher Teacher’s Training College, University of Yaoundé I, P.O. Box: 47, Yaoundé, Cameroon; 3 Department of Organic Chemistry, Faculty of Sciences, University of Yaoundé I, P.O. Box 812, Yaoundé, Cameroon

**Keywords:** Gastrointestinal infection, Anti-shigellosis, *Picralima nitida*

## Abstract

**Background:**

*Picralima nitida* Stapf (Apocynaceae) is a medicinal plant used traditionally in Cameroon to cure various ailments such as gastrointestinal disorders and dysentery. This study reports the *in vitro* and *in vivo* anti-shigellosis activity of the methanol extract of this plant on rats.

**Methods:**

The antimicrobial activity of the extract against pathogenic strains was evaluated using the disc diffusion assay and broth microdilution method. After oral administration of a suspension of *Shigella dysenteriae* type I (sd1), diarrheic rats were divided into 5 groups; the control group received the vehicle of the extract and the four others 125, 250, 500 mg/kg of the plant extract and ciprofloxacin (20 mg/kg) respectively for 7 days. The frequency of faeces emission as well as the weight of normal and diarrheic faeces was recorded. The presence of stools containing mucus or blood and the number of sd1 in faeces were also recorded.

**Results:**

*In vitro*, the extract had an antimicrobial effect on 11 out of the 17 pathogenic strains tested. The values of CMI and CMB obtain against *Shigella dysenteriae* type I were 800 and 6400 μg/ml respectively*. In vivo*, diarrhoea induction was effective and we notice an increase in faeces frequency and weight (p < 0.05), increase in the percentage of diarrheic stool released as well as the mucus contained in stool (p < 0.05), an increase in bacterial population in stool (p < 0.05). *Picralima nitida* extract, like ciprofloxacin markedly reduces the frequency faeces released and sd1 density from 100% (diarrheic rats) to 47.22 and 61.69% (500 mg/kg) respectively. It also slowed down the movement of charcoal meal through gastro-intestinal tract with the percentage of intestinal length covered of 60.54 (500 mg/kg).

**Conclusion:**

This anti-shigellosis activity *in vitro* and *in vivo* attests the usefulness of *Picralima nitida* in the traditional treatment of gastrointestinal disorders such as dysentery.

## Background

Shigellosis is an infectious disease caused by various species of *Shigella: S. boydii, S. dysenteriae, S. flexneri, and S. sonnei.* Out of these, *S. dysenteriae* is found worldwide and concentrated in areas where the population bear the brunt of overcrowding and malnutrition and do not possess adequate waste management and safe drinking water supplies [1]. Shigellosis is associated with 5-15% of cases of diarrhoea and 30-50% of cases of dysentery worldwide [2]. In malnourished children, shigellosis can cause a vicious cycle of further impaired nutrition absorption, recurrent infection, and growth retardation [3]. Without proper care, shigellosis can progress to the life-threatening systemic disease called hemolytic uremic syndrome, which is characterized by thrombocytopenia, hemolytic uremia, and kidney failure [4]. About 3% of persons who are infected with *S. flexneri* may subsequently develop pains in their joints, irritation of the eyes, and painful urination [5,6]. This condition called Reiter's syndrome can lead to chronic arthritis which is difficult to treat.

Shigellosis can usually be treated with antibiotics. Antibiotics commonly used are ampicillin, trimethoprim/sulfamethoxazole, nalidixic acid, the fluoroquinolone, ciprofloxacin. Some *Shigella* have become resistant to antibiotics and inappropriate use of antibiotics to treat shigellosis can make the organisms more resistant in the future. Resistance to quinolone antibiotics has been reported for *S. dysenteriae* type I (sd1) which rapidly develops resistance to the current therapy [7,8]. Dominant clone of ciprofloxacin–resistant sd1 was observed in Southeast Asia and in Canada [9,10]. This situation leads scientists to search for new sources of antimicrobial substances from various sources like medicinal plants [11,12].

*Picralima nitida* Stapf (PN) (Apocynaceae) is an entirely glabrous shrub of *3–10* m high*,* found in African forest region, spanning from Ivory Coast to Zaire and Uganda [13]. Medicinally, the plant is used in the treatment of malaria, male sexual impotence, dysmenorrhoea, gastrointestinal disorders and dysentery [13]. It has been shown to possess anti-trypanosomiac, anti-inflammatory, anti-pyretic and anti-plasmodial properties [14-17]. It has also been shown to possess an activity against Castor oil-induced diarrhea [18]. But the use of this medicinal plant as treatment against gastrointestinal infection as well as against dysentery is based simply on traditional beliefs perpetuated along several generations. The evaluation of the antidysenteric activity *in vitro* and *in vivo* of this medicinal plant will offer the best opportunity to understand its rational use in ethno-medicine.

This study reports the antimicrobial properties of methanol (M) extract of PN fruit-rinds against 17 pathogenic species involved in gastrointestinal infection isolated from patients as well as the evaluation of its anti-dysenteric activity as well as activity on gastrointestinal transit in rat model.

## Methods

### Plant material

The fruit of PN were collected in the morning in Zo-Etélé, Cameroon in September 2011. The identification of the plant was confirmed at the National Herbarium in Yaoundé-Cameroon (reference number of the plant: N° 2136/SRFK). The seeds of the fruits of PN were removed and the fruit-rinds were kept. The plant material (fruit-rinds of PN) was then air dried at room temperature. The dried plant material was ground into a fine powder.

### Chemicals and culture media

Mueller Hinton agar and salmonella-shigella agar were purchased from Oxoid, Basingstoke, England; Sabouraud dextrose agar from Merck SA, Sao Paulo, Brazil and Peptone water from HiMedia, Mumbai, India. Antimicrobial susceptibility testing was performed by the disc diffusion method with commercially available disks (Biomerieux F 69260 Charbonnières, France) for Cefoxitin (30 μg), Dibekacine (10 μg) and Clindamycin (10 μg); (Becton, Dickinson and Company Sparks, USA) for Ampicilin (10 μg), Colistin (10 μg), Nitrofurantoine (100 μg), Streptomycin (10 μg), Kanamycin (30 μg), Spectinomycin (25 μg) and Tobramycin (2 μg); (Oxoid, Basingstore, England) for Doxycyclin monohydrate (30 μg), Netilmicin (30 μg), Erythromicin (15 μg), Amoxycillin/Clavulanique (20/10 μg), Amoxicillin (10 μg), Gentamicin (10 μg), Vancomycin (30 μg); (Himedia, Mumbai, India) of ciprofloxacin (5 μg). Gentamicin (Gentamycin injection, 8 mg/2ml, ZMC Hamburg GMBH German), Nystatin (500000 UI, Novadina Pharmaceutical Ltd, London, United Kingdom) and Ciprofloxacin (ZOFLOX, ciprofloxacine 750 mg, Odypharm) used as reference antibiotics for the determination of the MIC and MBC as well as for the in vivo test were purchased from local pharmacy.

### Extraction

This was carried out by soaking the dried powdered plant (250 g) in bottle with 3.5 l of methanol (M) and kept for 72 h. The plant-(M) mixture was then sieved. The filtrate (extract) was concentrated by evaporating under vacuum (M) using a rotating evaporator. The extract was further concentrated by allowing it to stand overnight in an oven at 30°C. The yield of the extraction was calculated by dividing the weight of the extract obtained by the weight of the dried material plant extracted.

### Phytochemical screening

Preliminary analysis of chemical constituents such as alkaloids, leucoanthocyanins, flavonoids, saponins, tannins, anthraquinones, polyphenols, coumarins, sterols and/or triterpenes, anthocyanins, cardiac glycosides, reducing sugars, glycosides, anthranoids and steroids was carried out. Those constituents were identified by characteristic colour changes using standard procedures previously described [19]. Each test was qualitatively expressed as negative (−) or positive (+) and the intensity of the characteristic colour was expressed as (++) or (+++).

#### Test for alkaloids (Meyer reagent)

Fifty milligrams of the tested compound was heated in 10 ml of 2% H_2_SO_4_ for 2 min and filtered. To 1 ml of the filtrate, a few drops of the Meyer Reagent was added. The presence of alkaloids was indicated by the obtaining of a white precipitation or turbidity.

#### Test of flavonoides

Fifty milligrams of the tested compound was introduced in 5 ml of methanol. To it was added some magnesium chip and concentrated hydrochloric acid drop wise. The presence of flavonoids was indicated by the appearance of a violet or brick red coloration with effervescence.

#### Test for leucoanthocyanines

Fifty milligrams of the tested compound was dissolved in 10 ml distilled water. To 0.5 ml of this mixture was added 2 ml of 2N HCl and heated in a water bath at 100°C. The presence of leucoanthocyanines was indicated by the appearance of red coloration which later changes to violet.

#### Test for saponins

Fifty milligrams of the tested compound was added to 5 ml of distilled water. After homogenization, the mixture was boiled in 5 min, cooled and filtrated. 5 ml of the filtrate was vigorously agitated for 10 to 30 s. The presence of saponins was indicated by the appearance of foam which persists within 15 min.

#### Test for tanins

Fifty milligrams of the tested compound was added in 5 ml of distiller water. After mixing, the mixture was boiled for 5 min, cooled and filtrated. 5 ml of 2% NaCl was added to 10 ml of the filtrate and then filtered. The presence of tannins was indicated by the formation of a precipitate after the addition of 1% gelatin to the filtrate.

#### Test of anthraquinones

Fifty milligrams of the tested compound was diluted in 2 ml of chloroform, homogenized and filtered. Next, 1 ml of 10% NaOH was added to 1 ml of the filtrate. The presence of anthraquinone was indicated by the appearance of a red coloration.

#### Test of phenols and polyphenols

Fifty milligrams of the tested compound was dissolved in 15 ml distilled water, headed in a water bath for 15 min then cooled and filtered. To 2 ml of the filtrate was added drop wise 1 ml of 1% FeCl_3_ and 1 ml of 1% K_3_Fe(CN)_6_. The presence of polyphenols and phenols was indicated by the apparition of a blue and green precipitate respectively.

#### Tests of coumarines

On a thin layer chromatographic plate was deposited a spot of the tested compound dissolved in methanol. Next, the plate was exposed to ammoniac vapors. The presence of coumarines was indicated by the appearance of spot whose color varied from blue to purple in the presence of ammoniac.

#### Test of triterpenes and sterols (Liebermann- burchard test)

Fifty milligrams of the tested compound was dissolved in 2 ml of chloroform and next some drops of acetic anhydride added to the mixture and 1 ml of concentrated H_2_SO_4_. The presence of triterpenes was revealed by the apparition of violet-red coloration and sterols by a blue greenish coloration.

#### Test for anthocyanins

To 50 mg of the tested compound was added 15 ml of a concentrated HCl, the mixture was boiled. The presence of anthocyanins was indicated by the variation of coloration from orange red to orange blue during boiling.

#### Test for cardiotonic glycosides

In a test tube, 50 mg of the tested compound was dissolved in 2 ml of chloroform. 2 ml of the concentrated H_2_SO_4_ was introduced through the walls of the tube. Two phases were formed. The presence of cardiotonic glycosides was indicated by the observation of a brown ring at the interphase.

#### Test of reducing sugars

Fifty milligrams of the tested compound was dissolved in 10 ml of distilled water, mixed and filtered. In a conical flask containing 4 ml of a Fehling A and B (v/v) liquid mixture, was introduced drop wise of the filtrate. The mixture was boiled; the presence of reducing sugar was indicated by the appearance of a brick red coloration.

#### Test of glycosides

Fifty milligrams of the tested compound was added to 5 ml of 5% HCl then neutralized with 5 ml 5% NaOH. After homogenization, the mixture was filtered and Fehling liquid test was realized on the filtrate.

#### Test for anthranoides

Fifty milligrams of the tested compound was added to 2 ml of 0.5 KOH, then 0.5 ml of 5% H_2_O_2_ and boiled for 2 min. After cooling, the mixture was filtered. 6 drops of concentrated acetic acid and 3 ml of toluene added to the filtrate. The upper phase (toluene) was transferred to a test tube in which was added 1 ml of 0.5 N KOH. The presence of antranoides was indicated by the appearance of red coloration in the aqueous layer.

#### Test of steroids

Fifty milligrams of the tested compound was dissolved in 3 ml of chloroform, agitated intermittently for 2 hours and filtered. 1 ml of the filtrate was deposited on a porcelain plate. After evaporation, a drop of sulfuric acid was added, stirred and the color noted. To another portion of the filtrate was added a drop of acetic anhydride and the color noted. The reaction was positive if the same color was obtained for both sulfuric acid and acetic anhydride.

### Animals

Four weeks old *Wistar* albino rats (weighing 65–95 g) of both sexes were obtained from the animal house of the Centre for research in Food and Nutrition (CRAN) of the Institute of Medical Research and Medicinal Plant Studies (IMPM), Yaoundé. The rats were given food and water *ad libitum*. These animals were used for the antimicrobial drug assessment and the study of intestinal transit. Animal housing and the bioassay was conducted in accordance with the internationally accepted principle guidelines of the European Union on Animal Care (CEE Council 86/609). The Cameroon National Ethical Committee approved the protocols of the study (Ref n° FW-IRB 00001954).

### Microorganisms

The clinical microorganisms included here for the antimicrobial activity screening were the most common strains implicated in gastro-intestinal disorder such as diarrhea and dysentery. There were 15 bacterial and 2 yeast stains : *Escherichia coli LMP0101U (E. coli), Klebsiella pneumoniae LMP0210U (K. pneumoniae), Shigella dysenteriae type 1 LMP0504G (S. dysenteriae), Shigella flexneri LMP0313U (S. flexneri), Morganella morganii LMP0904G (M. morganii), Proteus vulgaris LMP0103U (P. vulgaris), Proteus mirabilis LMP0504G (P. mirabilis), Salmonella typhi LMP0209U (S. typhi), Citrobacter freundii LMP0804G (C. freundii), Enterobacter cloacae LMP1104G (E. cloacae), Enterobacter agglomerans LMP1004G (E. agglomerans), Staphylococcus aureus LMP0206U (S. aureus), Streptococcus feacalis LMP0207U (S. feacalis), Pseudomonas aeruginosa LMP0102U (P. aeruginosa), Bacillus cereus T* F 3748 *(B. cereus T), Candida albicans LMP0204U (C. albicans) and Candida glabrata LMP0205U (C. glabrata)*. These microorganisms were obtained from Bacteriology and Mycology Laboratories of Centre Pasteur of Yaoundé-Cameroon and *Bacillus cereus T* was obtained from the Microbiology Laboratory of the Institute of Food Research Reading of Great Britain.

### In vitro antimicrobial activity

#### Disc diffusion assay

The dried plant-extract was dissolved in the same solvent of extraction (M) to a final concentration of 125 mg/ml. Sterile paper disc (6 mm of diameter) prepared from Whatman number one filter paper were impregnated with 10 μl of the crude extract of the plant as described by Edward [20] from a stock of 125 mg/ml. Each disc contains 1.25 mg of the extract. These paper discs were kept in an incubator at 37°C for 24 h to evaporate the solvent. Antimicrobial tests were then carried out by disc diffusion method [21]. 100 μl of the suspension of the tested microorganism (0.5 Mc Farland standard turbidity) containing 10^8^ CFU/ml of bacterial, 10^6^ CFU/ml of yeast prepared from an overnight Mueller Hinton agar culture for bacterial and Sabouraud dextrose agar for yeast was used to seed each prepared and dried Mueller Hinton agar plate for bacteria and Sabouraud dextrose agar for yeast. The discs were arranged and firmly pressed on the agar surface of each seeded plate. These plates, after staying at 4°C for 2 h were incubated aerobically at 37°C for 24 h for bacteria and at 25°C for 24 h for yeast. Similarly, paper discs containing concentration of standard antibiotics (Gentamicin and Nystatin) were prepared and used for the susceptibility test. Negative control was also prepared using the same solvent employed to dissolve the plant extract. Antimicrobial activity was evaluated by measured the zone of inhibition against the tested microorganism and were expressed by – (negative), + (zone of inhibition ≤ 8 mm in diameter), ++ (zone of inhibition > 8 mm and ≤ 20 mm in diameter) and +++ (zone of inhibition > 20 mm in diameter). The result recorded for each bioassay was the average of 3 tests.

Only for *S. dysenteriae* type 1, a concentration dependent effect of the extract was evaluated using disc diffusion method described above. The reason of this choice was that the strain will later be used to induce bacillary dysentery. The concentrations of the extract tested were 5, 2.5, 1.25 and 0.625 mg. In order to determine their multi-drug resistance profiles, antimicrobial susceptibility testing was performed also against Sd1 by the same method with commercially available disks of Gentamycin 10 μg, Amoxicyclin/clavulanic Acid 20/10 μg, Nitrofurantoin 100 μg, Kanamycin 30 μg, Ampicilin 10 μg, Netilmicin 30 μg, Colistin 10 μg, Erythromycin 15 μg, Ciprofloxacin 5 μg, Amoxycylin 10 μg, Vancomycin 30 μg, Doxycyclin monohydrate 30 μg, Spectinomycin 25 μg, Tobramycin 2 μg, Clindamycin 10 μg, Dibekacin 10 μg, Steptomycin 10 μg and Cefoxitin 30 μg.

#### Determination of MIC and MBC

Minimum inhibitory concentration (MIC) and minimum bactericidal concentration (MBC) were assessed for the microorganisms that were determined as sensitive to (M) extract of PN in disc diffusion assay using the broth microdilution method [22]. The test was performed in peptone water supplemented with glucose 1% (w/v) with red phenol as a colour indicator (PPG1%). Bacterial strains were cultured overnight at 37°C in Muller Hinton and yeast strains at 25°C in Sabouraud dextrose agar. Test strains were suspended in normal saline (NaCl 9‰), adjusted to 0.5 Mc Farland standard turbidity and suspended in PPG1% to give a final density of 5×10^5^ CFU/ml. For the susceptibility test, the 96-well round bottom sterile plates were prepared by dispensing 180 μl of each inoculated broth into wells. A 20 μl aliquot of the plant extract was added. The concentration of extract used to evaluate the antimicrobial activity was included from 0.39 to 12800 μg/ml. One well was considered as growth control since no extract solution was added. The final volume of each well was 200 μl. The content of each well was mixed and then incubated under normal atmospheric condition at 37°C for 24 h (for bacterial strains) and at 25°C for 24 h (for yeasts strains).

The bacterial and yeasts growth was indicated by the colour change of the well content from the red to yellow. The MIC was defined as the lowest concentration of the extract to inhibit the growth of microorganism. MBC were determined by platting 5 μl sample from red wells on Mueller Hinton agar or on Sabouraud dextrose agar without extract. The MBC was the concentration at which there was not microbial growth. The extract tested in this study was screened three times against each microorganism. Gentamicin for bacteria strains and Nystatin for yeast strains at the concentration range of 0.019 to 320 μg/ml was prepared in (PPG1%) and used as standard drugs for the positive control. The susceptibility of the sd1 to antibiotic Ciprofloxacin which shown the great activity against sd1 in disc diffusion assay was also performed by liquid dilution method and served as positive control.

### In vivo antimicrobial activity

Thirty rats were individually kept in metabolic cage during a period of observation of 7 days before diarrheal induction. Diarrhoea was induced in rats using sd1 strains. The turbidity of the sd1 inoculum was matched spectrophotometrically at 450 nm to 4 McFarland standards. After verifying that the rats were not sd1 carriers, they were orally administrated with 1 ml of the saline diluted inoculum. Diarrheic rats were randomly divided into 5 groups, each containing six animals. When diarrhoea appeared, animal were administrated the anti-diarrheic drugs daily by the oral route for 7 consecutive days: The first group (diarrheic control) received the vehicle of the extract (0.5% Tween 80 in distilled water); the second group received the antibiotic ciprofloxacin (20 mg/kg of body weight (bw) and the remaining three groups were given orally 125, 250, and 500 mg/kg bw of M extract of PN.

Animals were observed daily for behavioural changes and mortality patterns once before induction, during induction and up to 7 days after induction. The stools of each animal were also collected daily using a white cloth fixed under the bars supporting the animal in the metabolic cage during all the experiment period. Then, the frequency and weight of normal and diarrheic faeces were recorded. The presence of stools containing mucus or blood was also noted daily at each experimental period. Enumeration of sd1 in faeces was performed before diarrheal induction to make sure that the rats are not sd1 carriers, after the appearance of diarrhoea and once every day during the 7 days of treatment. For this purpose, 0.5 mg of faeces was homogenized in 4.5 ml of sterile saline; serial of dilution were made and 50 μl of each dilution was seeded over salmonella-shigella agar. After 24 h of incubation at 37°C, the number of CFU was determined.

### Study of small intestinal transit

This was done according to the method proposed by Mujumdar [23] using charcoal meal as a diet marker. The rats were divided into 4 groups of 6 animals each. The first group (the control group) was orally administered the vehicle. The second and third groups orally received M extract of PN, 250 mg/kg and 500 mg/kg bw respectively. The fourth group also orally received the standard drug, atropine sulphate (5 mg/kg bw). 30 min after administration, each animal was given 1 ml of charcoal meal orally (10% activated charcoal in 5% gum acacia). Also, 30 min after this administration, each animal was sacrificed and the distance covered by the charcoal meal in the intestine, from the pylorus to the caecum was measured and expressed as a percentage of distance moved.

#### Statistical analysis

The results are expressed as means ± standard deviation. Bacterial densities were expressed in log10 before analysis. Data were statistically evaluated using the analysis of variance following by the paired t-student’s test. The differences between groups were considered significant at p < 0.05.

## Result

### Phytochemical screening

The yield of the extraction was 5% w/w. Phytochemically analysis revealed the presence of alkaloids, flavonoids, saponins, polyphenols, cardiac glycosides and glycosides in which alkaloids, and polyphenols were major compounds (Table 1).

**Table 1:** **Chemical composition of methanol extract of*Picralima nitida*fruit-rinds**

**Chemical compounds**	**Observations**
Alkaloids	+++
Leucoanthocyanins	─
Saponins	++
Tannins	+
Antraquinones	–
Polyphenols	+++
Coumarins	–
Sterols and/or Triterpenes	–
Cardiac Glycoside	++
Reducing sugars	+
Glycosides	++
Anthranoid	–
Steroids	+
Flavonoids	++
Anthocyanin	–

(−): Absence of chemical compound (+): Presence of chemical compound.

(+)< (++) < (+++): Base on the intensity of characteristic colour.

### In vitro antimicrobial activity

The antimicrobial activity of the fruit-rinds of PN extracts against microorganisms examined in the present study and their potency were qualitatively and quantitatively assessed by the presence or absence of inhibition zone and zone diameter, MIC and MBC values. The results are given in the Tables 2 and 3.

**Table 2:** **Antimicrobial activity of the (M) extract of PN (fruit-rinds) against microorganisms tested using*Disc diffusion assay*(1.25 mg of the extract/disc)**

**Microorganisms**	***M.m***	***E.c***	***S.a***	***P.V***	***C.f***	***S.f***	***S.d***	***E.c***	***E.a***	***S.fe***	***P.a***	***P.m***	***S.t***	***K.p***	***B.c***	***C.a***	***C.g***
PN -M	–	++	++	+++	–	–	++	++	–	++	++	++	++	–	++	++	–
Gentamicin	++	++	+++	+++	++	++	++	+++	++	++	+++	+++	++	++	++	NT	NT
Nystatin	NT	NT	NT	NT	NT	NT	NT	NT	NT	NT	NT	NT	NT	NT	NT	++	++

*M.m: M.morganii; E.c: E coli; S.a: S.aureus; P.V: P.vulgaris; C.f: C.freundii; S.f: S.flexneri; S.d: S.dysenteriae; E. c: E.cloacae; E.a: E.agglomerans; S.fe: S.feacalis; P.a: P.aeruginosa; P.m: P.mirabilis; S.t: S.typhi; K.p: K.pneumoniae; B.c: B.cereus; C.a: C.albicans; C.g: C.glabrata.* NT: Not tested*.* (+): Zone of inhibition ≤ 8 mm in diameter. (++): Zone of inhibition > 8  mm and ≤ 20 mm in diameter. (+++): Zone of inhibition > 20 mm in diameter. (−): Negative.

**Table 3:** The MIC and MBC values of the (M) extract of PN (fruit-rinds) against the microorganisms tested using microdilution assay

**Microorganisms**	***P.a***	***S.fe***	***En.c***	***S.d***	***P.v***	***E.c***	***B.c***	***S.a***	***S.t***	***P.m***	***C.g***	***C.a***
MIC (μg/ml)	50	200	>12800	800	0. 78	3.12	200	3.12	100	100	25	>12800
MBC(μg/ml)	1600	1600	>12800	6400	1.56	12.5	800	12.5	400	400	200	>12800

Values are the mean of three independent determination; the variation was less than 1/100 000. *E.c: E coli; S.a: S.aureus; P.V: P.vulgaris; S.d: S.dysenteriae; En. c: E.cloacae; S.fe: S.feacalis; P.a: P.aeruginosa; P.m: P.mirabilis; S.t: S.typhi; B.c: B.cereus; C.a: C.albicans; C.g: C.glabrata.* MIC: minimal inhibition concentration (μg/ml). MBC: minimal bactericidal concentration (μg/ml).

The (M) extract of PN had antibacterial effect on 11 out of 17 rats involved in either diarrhea, dysentery or other gastrointestinal disorders (Table 2). The antimicrobial effect of this extract was found to be comparable to that of the conventional antibacterial (Gentamycin) and antifungal (Nystatin) drug used in this study. Maximal inhibition zone values for the microorganisms sensitive to the (M) extract of PN was 21.83 mm against the Gram-negative bacterium *P. vulgaris* (Table 2).

Considering that in this study crude extract was employed, the MIC values of 8 mg/ml or below against any microorganism tested was considered as active. The best MIC and MBC values for the microorganisms tested were 0.78 and 1.525 μg/ml respectively (Table 3). The greater and remarkable antimicrobial activity of the extract was recorded with *P. vulgaris.* Antimicrobial activity of the extract of PN was least observed with *C. albicans* and *E. cloacae,* the two fungal strains used.

A concentration dependent effect of the extract against sd1 was observed with agar diffusion method. The inhibition diameter began at 0.625 mg (Ø= 8.33 mm) and increase progressively up to 24.5 mm for 5 mg. The result of the antimicrobial susceptibility test performed with commercially available discs has revealed that sd1 strain included here was resistant to Kanamycin, Tobramycin, Clindamycin, Amoxicyclin/Clavulanic acid, Amoxycylin, Cefoxitin and Ampicilin (Table 4). The highest strain sensitivity was obtained with Ciprofloxacin and was confirmed by data from literature [24]. The values of CMI and CMB obtained were 800 and 6400 and 0.07 and 0.312 μg/ml for PN and Ciprofloxacin respectively.

**Table 4:** Antimicrobial susceptibility test performed with commercially available disks using Disc diffusion assay

**Antibiotics**	**G**	**A/C**	**Ni**	**K**	**A**	**N**	**C**	**E**	**C**	**A**	**V**	**Dm**	**S**	**T**	**C**	**D**	**S**	**Ce**
Diameters of the inhibitory zones (mm)	18	0	14	0	0	28	14	12	24	0	18	12	12	0	0	18	18	0

Gentamycin (G), Amoxicyclin/ clavulanic Acid (A/C), Nitrofurantoin (Ni), Kanamycin (K), Ampicilin (A), Netilmicin (N), Colistin (C), Erythromycin (E), Ciprofloxacin (C), Amoxycylin (A), Vancomycin (V), Doxycyclin monohydrate (Dm), Spectinomycin (S), Tobramycin (T), Clindamycin (C), Dibekacin (D), Steptomycin (S) Cefoxitin (Ce).

### In vivo antimicrobial activity

#### Animal behaviour, stool quality and mortality

The animals showed behavioural changes 3 hours after the administration of the inoculum. The changes which lasted 2 to 3 days of treatment, included lethargy, disorientation, seizures. The first diarrheic stool was emitted within 24 hours after the induction. Diarrheic stools were either soft or liquid, containing mucus and smooth and very often mucus-linked. During the 7 days of treatment, no death was observed, except in the groups of animal receiving the vehicle and the dose of 125 mg of the extract where 83.33 and 50% of deaths were respectively registered at the end of the experimental period (Table 5).

**Table 5:** **Effect of the treatment on the mortality of*shigella dysenteriae*type 1 diarrheic rats**

**Day after induction**	**Number of death**				
	**Diarrheic control**	**Ciprofloxacin**	***Picralima nitida***		
			**125 mg/kg**	**250 mg/kg**	**500 mg/kg**
1	0	0	0	0	0
2	0	0	0	0	0
3	0	0	0	0	0
4	1	0	0	0	0
5	1	0	1	0	0
6	1	0	1	0	0
7	2	0	1	0	0

#### Stool bacterial density

**Figure 1:**
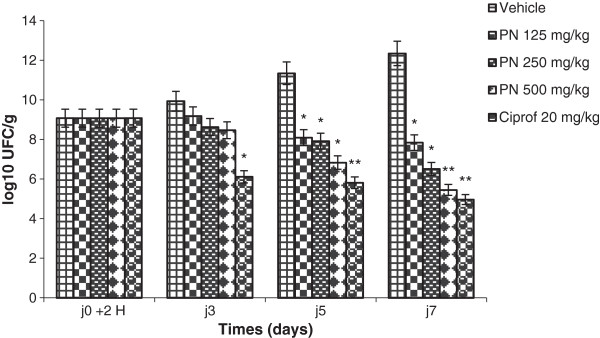
***Shigella dysenteriae *type I density in stool (log**_**10 **_**UFC/g) over 7 days of treatment with methanol extract of *Picrilima nitida *(PN) and ciprofloxacin (Ciprof).** Each data column represents the mean ± S.E.M. (n = 6). Data column of the same day with superscript * are significantly different compared with diarrheic control (* p < 0.05; **p < 0.01).

In the stools of diarrheic control rats, sd1 density increased significantly from the first day after induction to the last day of the treatment compared with the initial value administered. Compared with the diarrheic control and the initial value administered, the antibiotic ciprofloxacin significantly reduced the density of sd1 in the stool from the first to the last day of the treatment. Similar to ciprofloxacin, PN inhibited the bacterial growth in a dose depended manner. PN extract at the dose 125 mg/kg inhibited bacterial growth by the 3^rd^ day but maintaining the sd1 density at a level slightly inferior to the value administered. The extract at the dose of 250 and 500 mg/kg effectively reduced sd1 density from the 3^rd^ day of therapy and beyond; the percentage of reduction of sd1 density was respectively 61.69 and 65.67% compared to the value administrated (Figure 1).

#### Frequency and mass of normal and abnormal stool

**Figure 2:**
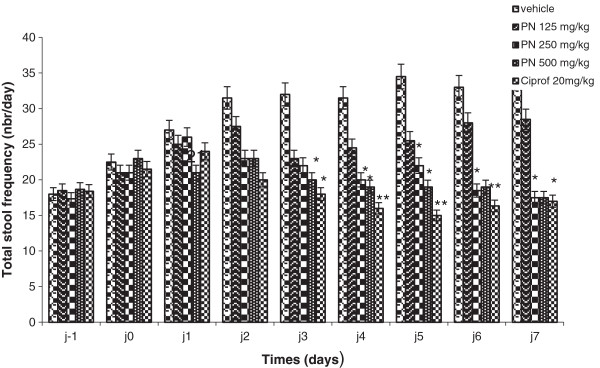
**Total stool frequency before and during treatment of *Shigella dysenteriae *type I diarrheic rats with methanol extract of *Picrilima nitida *(PN) and ciprofloxacin (Ciprof).** Each data column represents the mean ± S.E.M. (n = 6). Data column of the same day with superscript * are significantly different compared with diarrheic control (* p < 0.05; **p < 0.01).

**Figure 3:**
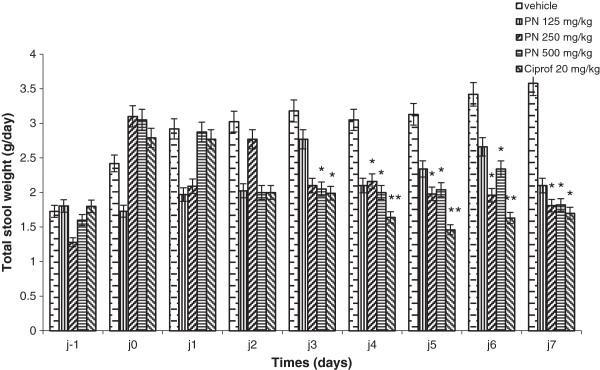
**Total stool weight before and during treatment of *Shigella dysenteriae *type I diarrheic rats with methanol extract of *Picrilima nitida *(PN) and ciprofloxacin (Ciprof).** Each data column represents the mean ± S.E.M. (n = 6). Data column of the same day with superscript * are significantly different compared with diarrheic control (* p < 0.05; **p < 0.01).

The study shows that standard drug and the extract markedly reduced the frequency of faeces released from 100% (diarrheic rats) to 47.22 and 79.16 (125 mg/kg) to 48.61% (250 and 500 mg/kg) respectively. From the 2^nd^ day of the treatment, the total faeces frequency, while increasing in control diarrheic rats, decreased in all the treated rats. This reduction was significant from the 3^rd^ day in rats receiving standard drug, 250 and 500 mg/kg of the extract. The average weight of defecate in the control group at the end of the treatment was 3.58 g. Treatment with each dose of the medicinal plant extract significantly reduced the weight of the defecation to 41.35 (125 mg/kg) - 49.35% (500 mg/kg) compared with control group (Figures 2 and 3).

The rats that did not receive the medicinal plant extract or standard drug showed typical symptoms of shigellosis along with watery, frequent defecation and frequent mucous stools. The percentage of diarrheic and mucus contained in stools of the survival animal in the control group were respectively 95.238 and 61.90% at the end of the treatment. Treatment with each dose of the medicinal plant extract significantly reduced the percentage of diarrheic stools to 38.88 (125 mg/kg) – 0.00% (500 mg/kg) compared with control group. Like diarrheic stool, mucus contained in stool also reduced with the treatment. The percentage of mucus contained in stool were 1.00 (125 mg/kg) to 0.00% (250 and 500 mg/kg) at the end of the treatment (Tables 6 and 7).

**Table 6:** **Effect of the treatment on the percentage (%) of diarrheic stool of*shigella dysenteriae*type 1 diarrheic rats**

**Days**	**j0**	**j1**	**j2**	**j3**	**j4**	**j5**	**j6**	**j7**
Vehicle	0	5.55	25.40	32.79	56.60	72.00	80.95	95.23
PN (125 mg/kg)	0	4	10.34	19.05	20.00	23.26	26.09	38.88
PN (250 mg/kg)	0	2.24	4.35	3.85	0	0	1.22	0
PN (500 mg/kg)	0	3.57	2.32	1.75	0	0	0	0
Ciprof (20 mg/kg)	0	3.70	1.75	0	0	0	0	0

**Table 7:** **Effect of the treatment on the percentage (%) of mucus contains stool of*shigella dysenteriae*type 1 diarrheic rats**

**Days**	**j0**	**j1**	**j2**	**j3**	**j4**	**j5**	**j6**	**j7**
Vehicle	0	14.76	19.05	16.39	22.64	48.00	57.14	61.90
PN (125 mg/kg)	0	4.00	6.90	13.04	12.22	14.82	9.302	2.32
PN (250 mg/kg)	0	2.32	0	0	0	0	1.04	0
PN (500 mg/kg)	0	3.57	0	0	0	0	0	0
Ciprof (20 mg/kg)	0	0	0	0	0	0	0	0

#### Effect of PN-M on charcoal-induced gut in transit changes

The administration of PN-M also slowed down the movement of charcoal meal through gastro-intestinal tract compared to the castor oil-treated rats. The percentage of intestinal length covered by charcoal meal in PN-M pre-treated (250 and 500 mg/kg) and castor oil-treated rats was 58.27, 60.54 and 74.57 respectively. Atropine on its part, produced a marked decrease in the propulsive movement and the intestinal length covered by charcoal meal was 40.33 (Table 8).

**Table 8:** Effect of PN-M on charcoal-induced gut transit changes

**Group**	**Percentage of distance travelled by charcoal meal**	**Percentage of inhibition of the propulsive effect (%)**
Vehicle	74.57 ± 9.90	0
Atropine sulphate (5 mg/kg)	40.32 ± 4.25^**^	45.93
PN (250 mg/kg)	58.27 ± 11.20^*^	21.86
PN (500 mg/kg)	60.54 ± 8.17^*^	18.81

Data are means ± standard deviation (n = 6 per group). Values in the same column with superscript * are significantly different compared with control group (* p < 0.05; **p < 0.01).

## Discussion

The (M) extract of PN fruit-rinds showed a promising broad spectrum antibacterial property, inhibiting 80% of bacterial strains tested, especially *P. vulgaris, E. coli and S. aureus* with MBC ranging between 0.78 and 1.56 μg/ml. The presence of flavonoids, saponins, tannins and alkaloids in this extract as shown in Table 1 may explain some of its antimicrobial action since antimicrobial action of most of these phytochemical substances has been demonstrated [25-28]. These results are interesting since these microorganisms are commonly involved in acute diarrhoea diseases. Our data confirm previous results about antimicrobial activity of this plant. It has been shown that other parts (seed, root and stem bark) of *Picralima nitida* has significant antimicrobial activity [29,30]. To the best of our knowledge, this is the first time that the antimicrobial activity of the fruit-rind of this plant is reported.

The extract shows a concentration dependent effect against sd1. This inhibiting activity of the extract against sd1 was also confirmed by the sd1 count in stools, where the decrease of bacterial population was observed by the 3^rd^ and 5^th^ day of treatment with both extract and standard antibiotic used. Ciprofloxacin is a bactericidal synthetic antibiotic belonging to fluoroquinolone family. It has been recognized to have a great activity against sd1. Our data confirms this assertion since the great sensitivity of sd1 was observed with ciprofloxacin on the 18 standard antibiotics used. It acts by inhibiting the activity of DNA gyrase and thereby preventing the supercoiling of the bacterial chromosome [31]. Consequently, the bacterial can no longer pack its DNA into the cell.

*S. dysenteriae* causes dysentery by invading the colonic mucosa. Following host epithelial cell invasion and penetration of the colonic mucosa, *Shigella* infection is characterized by degeneration of the epithelium and inflammation of the lamina propria. This results in desquamation and ulceration of the mucosa, and subsequent leakage of blood, inflammatory elements and mucus into the intestinal lumen [1]. Patients suffering from *Shigella* infection will therefore pass frequent, scanty, dysenteric stool mixed with blood and mucus, since, under these conditions, the absorption of water by the colon is inhibited. Many diarrheic rats showed typical symptoms of shigellosis, along with watery, frequent defecation and frequent mucoid stools. Abnormalities of the central nervous system including lethargy, disorientation, seizures and paralysis were also observed within those animals. The multiplication of sd1 within colonic epithelial cells, causing cell death, and spreading laterally to infect and kill adjacent epithelial cells was suspected to being responsible for those typical signs that occurred within 2 days after induction. In addition, the signs of neurotoxicity and deaths observed within diarrheic animals may be due to shiga-toxin since it has been documented [32]. Symptoms of shigellosis in rats reduced significantly with both Ciprofloxacin and extract treatment with respect to the reduction of sd1 density in stool. This result confirms the antimicrobial activity of the extract *in vitro* and *vivo*.

PN-M significantly reduced intestinal transit as observed by the decrease in intestinal motility of charcoal meal. Probably extract increased the reabsorption of water thereby decreasing intestinal motility as observed by the decrease in intestinal transit of charcoal meal. The delay in faecal emptying by the extract allows more time for fluid absorption and subsequently reduces fluid loss in the stool. Decrease in intestinal transit induce by the extract could have lead to an increase of enteroinvasive infections, but this might have been prevented by the antimicrobial properties of the extract. Earlier studies have shown that anti-dysenteric and anti-diarrhoeal properties of medicinal plants were found to be due to tannins, alkaloids, saponins, flavonoids, sterol and/or triterpenes and reducing sugars [33,34]. Thus, alkaloids, saponins and flavonoids content of this plant extract may be responsible for its anti-diarrhoeal property.

## Conclusion

*S. dysenteria* type 1 strain was resistant to all antibiotics from the beta-lactam family used in this study. The great sensibility of the strain was obtained with ciprofloxacin, a synthetic antibiotic belonging to fluoroquinolone family. The (M) extract of PN had antimicrobial activity on 11 out of 17 microorganisms tested amongst witch *S. dysenteriae* type 1. The values of CMI and CMB obtain against this strain were 800 and 6400 μg/ml respectively*.* This anti-*Shigella* activity of PN was also shown *in vivo*. The administration of PN extract to diarrheic rats significantly reduced the bacterial population in stools; thereby reduce the symptoms of shigellosis on rats.

At present, our group is concerned with the fractionation and the isolation of pure compounds of the crude extracts and the elucidation of their structures in order to better evaluate their pharmacological activity *in vitro* and *in vivo*.

## Competing interests

The authors declare that they have no competing interests.

## Authors’ contributions

JK conducted experiments on phytochemical screening and LBKM on antimicrobial and anti-shigellosis activity of tested plant extract. JLT participated in design of the study and preparation of the manuscript. All the authors read and approved the final manuscript.

## Pre-publication history

The pre-publication history for this paper can be accessed here:

http://www.biomedcentral.com/1472-6882/13/211/prepub
